# Whole Exome Sequencing Reveals a *BSCL2* Mutation Causing Progressive Encephalopathy with Lipodystrophy (PELD) in an Iranian Pediatric Patient

**DOI:** 10.22045/ibj.2016.07

**Published:** 2016-11

**Authors:** Mohammad Reza Alaei, Saeed Talebi, Mohammad Ghofrani, Mohsen Taghizadeh, Mohammad Keramatipour

**Affiliations:** 1 Department of Pediatric Endocrinology, Faculty of Medicine, Shahid Beheshti University of Medical Sciences, Tehran, Iran; 2 Department of Medical Genetics, School of Medicine, Tehran University of Medical Sciences, Tehran, Iran

**Keywords:** Exome, *BSCL2*, Seipin, Iran

## Abstract

**Background:**

Progressive encephalopathy with or without lipodystrophy is a rare autosomal recessive childhood-onset seipin-associated neurodegenerative syndrome, leading to developmental regression of motor and cognitive skills. In this study, we introduce a patient with developmental regression and autism. The causative mutation was found by exome sequencing.

**Methods:**

The proband showed a generalized hypertonia and regression of all developmental milestones. Based on the advantages of next-generation sequencing (NGS), whole exome sequencing (WES) was requested. The functional significance of variants was evaluated by NGS-specific prediction servers. Sanger sequencing was used for segregation analysis in the family.

**Results:**

There was no specific sign in the clinical and paraclinical investigations of the patient to establish a conclusive clinical diagnosis. WES detected a known homozygous nonsense mutation in *BSCL2* (NM_001122955.3:c. 985C>T; p.Arg329*). The variant is segregating in the pedigree with an autosomal recessive pattern.

**Conclusion:**

Exome sequencing is a robust method for identifying the candidate gene variants in Mendelian traits.

## INTRODUCTION

*BSCL2* (Berardinelli-Seip congenital lipo-dystrophy 2; OMIM: 606158) was first identified in BSCL2 patients in 2001 [1]. The gene, which is also called seipin, is located on chromosome 11q13 and contains 11 exons. It has been revealed through Northern blot analysis that *BSCL2*/seipin produces three transcripts of 2.2 kb, 1.8 and 1.6 kb. The 1.8-kb mRNA is only expressed in brain and testis but the two other transcripts are expressed ubiquitously[2].

Seipin protein has three isoforms (1-3) that are 462, 398, and 287 amino acids long, respectively[3]. All three isoforms contain two hydrophobic amino acid regions predicted to be transmembrane domains. Seipin can anchor to membranes through hydrophobic interactions using these regions. In fact, seipin resides in endoplasmic reticulum where these two trans-membrane domains are buried in endoplasmic reticulum membrane. The middle part of the protein, which is conserved during evolution, is located inside the endoplasmic reticulum lumen, and both N-terminal and C-terminal domains face cytoplasm[2,4-7]. Although the function of seipin is not completely elucidated, some researchers have shown that the protein has a role in adipogenesis, biogenesis of lipid droplets and metabolism of lipids[8-14]. However, others have depicted a potential neural involvement[15-18].

Mutations in *BSCL2*/seipin cause two distinct phenotypes. Loss-of-function mutations are responsible for congenital generalized lipodystrophy type 2 (Berardinelli-Seip syndrome type 2; OMIM: 269700). Gain-of-function mutations or gain-of-toxic function mutations in seipin gene result in neurological disorders like Silver syndrome/spastic paraplegia 17 (OMIM: 270685) and distal hereditary motor neuropathy type V (OMIM: 600794). These disorders are currently being referred to as “seipinopathies”. Patients with seipinopathies have heterogeneous symptoms and manifest both upper and lower motor neuron disruptions[19].

Recently, a new seipin-associated neurodegenerative syndrome has been introduced by Guillén-Navarro *et al*.[20], known as progressive encephalopathy with or without lipodystrophy (PELD; OMIM: 615924). They found six children affected with severe, progressive encephalopathy from four apparently unrelated families. All of the patients were from Murica in southeastern Spain, and the transmission pattern of the disorder was compatible with autosomal recessive inheritance.

Whole exome sequencing (WES) is an innovative exome-targeted technique that utilizes sequence capture technology to selectively capture the exome region within the whole genome, followed by target fragments enrichment and high-throughput sequencing. Here, we present an Iranian patient carrying the R329X mutation in the *BSCL2*, whose diagnosis was difficult, and finally clinical WES confirms the diagnosis of PELD.

## MATERIALS AND METHODS

### Subjects and clinical assessment

The research was reviewed and approved by a duly constituted Ethics Committee of Tehran University of Medical Sciences (Tehran, Iran). A written informed consent was obtained from parents of the patient. Blood samples from the proband and his parents were collected and processed. Clinical evaluations included standard history, physical examination, brain MRI, brain CT-Scan, and metabolic profiling.

### DNA extraction

Genomic DNA was extracted from the blood leucocytes of the proband and his parents using the QIAamp blood kit (QIAGEN, Hilden, Germany) according to the manufacturer’s protocols.

### Whole exome sequencing

WES was performed by BGI Shenzhen (Beijing Genome Institute, Shenzhen, China). Exons of DNA samples were captured using the in-solution SureSelect Target Enrichment System (Agilent, Human All Exon Kits v2; Agilent Technologies, Inc., Santa Clara, CA, USA), followed by a paired-end high-throughput sequencing on reads of 75 bp using Illumina HiSeq 2000 (Illumine Inc., San Diego, CA, USA). A 23-giga base sequence was generated with at least 98.08% coverage for 4×, 91.86% for 20×, and 85.66% for 30× of the sample. The coverage of the target region was 98.93%, and the mean depth was 90.58×. Sanger sequencing was performed to confirm the candidate variants found in WES as well as segregation analysis of the candidate variants within the family.

### *In silico* pathogenicity assessment of variants

Non-exonic and synonymous variants were removed. The process was followed by the removal of common variants (i.e., minor allele frequency >0.02) reported in the single nucleotide polymorphism database (dbSNP), the 1-k human genome, the ESP6500 and BGI in house databases. To evaluate the pathogenicity of the novel variants, we analyzed the potential impact of a given variant on the function or structure of the encoded protein. The analysis was carried out based on conservation, physical properties of the amino acids or possible occurrence in regulatory or splicing motifs using bioinformatic tools SIFT (sift.jcvi.org), PolyPhen-2 (http://genetics.bwh.harvard.edu/pph2), Combined Annotation Dependent Depletion[21], and MutationTaster[22]. Among the prioritized variants, truncating mutations or mutations predicted to be damaging were considered to be the most promising candidates. PubMed and OMIM were reviewed for previous publications regarding candidate genes as well as functional and expression data.

## RESULTS

### Clinical findings

**Fig. 1:**
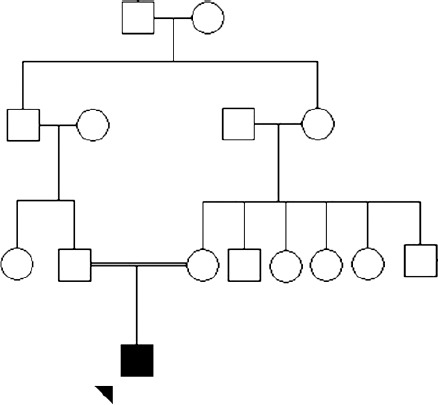
Pedigree of the Iranian family with Progressive encephalopathy with or without lipodystrophy.

The proband was a boy born at term via cesarean section (due to fetal distress) from a 25-year-old mother. The parents were first-degree cousins. Family history was unremarkable for genetic or metabolic disorders (Fig. 1). His birth measurements (weight: 3000 g, head circumference: 36 cm and length: 49 cm) and the neonatal history were normal. Due to poor weight gain at early infancy, soy-based, lactose free formula was added to the breast feeding. He was operated for inguinal hernia at 4^th^ month of age and was hospitalized one more time due to pneumonia at 6^th^ month of age. His growth was within the acceptable range. The motor and cognitive developmental milestones were within the acceptable range until two years but the speech and social skills were delayed. At 2.5 years of age, ritalin was prescribed for hyperactivity. Afterwards, other developmental milestones, especially motor skills, regressed significantly. When ritalin was discontinued, his skills improved slightly but the regression continued later.

Electroencephalography demonstrated the presence of non-convulsive status epilepticus, which was intractable to anti-epileptic drugs. Convulsive epilepsy appeared later. His weight gain was poor but the height growth was normal (weight: 14 kg and height: 117 cm at 7.5 years of age). He had a history of a dry skin with excessive sweating of hand and palm. There was a generalized loss of skin fat, especially in the facial skin. In addition, there was a generalized hypertrichosis with low frontal hairline. The neurological examination showed an autistic child with repetitive and stereotypic hand movements, repetitive upward staring, ataxia, generalized hypertonia, and severe global developmental delay. Fundoscopic examination was normal. However, the death occurred at the age of eight following an episode of status epilepticus.

The brain CT-Scan and MRI in the 3^rd^ year of age showed only an arachnoid cyst in the left hippocampus. Evaluation of plasma biochemical metabolites, including glucose, calcium, urea, creatinine, ammonia, lactate, blood gas, aminoacid profile, and acylcarnitine profile demonstrated no pathologic finding. Liver and thyroid function tests were also normal.

### Genetic findings

Totally, 131811 SNPs and 17317 indel variations were detected in the exome analysis (Table 1). To identify the pathogenic mutation, the exome data sequence variants were filtered step-by-step to reduce the number of potentially pathogenic variants as described above. Finally, we sorted the filtered variants according to the zygocity and Combined Annotation Dependent Depletion-PHRED score (Cut-off=15). Seven homozygous variants were found in genes like *BSCL2*, *CEP164*, *HPS5*, *PEX16*, and *CHRNA3* (Table 2). Only the mutation in the *BSCL2*, which causes PELD syndrome, was compatible with the clinical findings of the proband. The *BSCL2* mutation was a nonsense mutation in exon 7 of the gene (NM_001122955.3:c.985C>T[p.R329X]).

**Table 1:** Summary of variants detected through whole exome sequencing

Variant types	Proband
Total number of variants obtained	149128
Total Indel variants	17317
Exonic non-synonymous variants*	226
Exonic non-synonymous homozygous variants*	14
Coding Indel*	22
Frame shift*	3
Nonsense*	1
Splice site*	1

* Minor allele frequency (MAF)<0.02

**Table 2:** Exonic homozygous variants (MAF<0.02) with high CADD score

Gene name	Transcript*	DNA change	Amino Acid change	Fr. 1	Fr. 2	Fr. 3	Fr. 4	Raw score	PHRED v1.3
*BSCL2*	NM_001122955.3	c.985C>T	p.Arg329*	.	.	.	0	9.94193	36
*CEP164*	NM_014956.4	c.1246C>T	p.Arg416Cys	.	.	.	0	5.40362	26
*HPS5*	NM_181507.1	c.2866T>C	p.Tyr956His	0.000693	.	0.000693	0	5.0166	25.2
*HPS5*	NM_181507.1	c.1685C>T	p.Thr562Met	0.0005	0.0005	.	0.0002	2.18428	17.41
*PEX16*	NM_057174.2	c.760G>C	p.Val254Leu	0.014096	0.0101	0.018712	0.0101	1.98294	16.1
*DDHD1*	NM_001160148.1	c.336_337insGGCGGC	p.Gly112_Ser113insGlyGly	0	.	.	.	1.95542	15.93
*CHRNA3*	NM_000743.4	c.67_69delCTG	p.Leu23 del	0	.	.	.	1.88124	15.47

*All rare variants in OMIM disease (less than 2% in 1k human genome, dbSNP, ESP6500 or BGI in house database) are listed in this table. *The report is based on OMIM database updated on 19 January 2015. *Transcript, the ID of mRNA transcript of the gene. Mostly, we choose the longest one; Fr.1, dbSNP allele frequency; Fr.2, 1K-genome database frequency; Fr.3, ESP6500 frequency; Fr.4, BGI in-house database frequency. MAF: minor allele frequency, CADD: Combined Annotation Dependent Depletion.

**Fig. 2:**
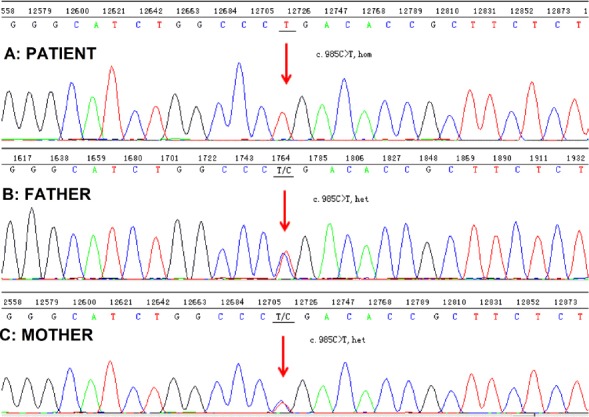
Sequencing chromatograms of the proband and his parents.

MutationTaster predicts this variant to be disease causing. This mutation was not present in dbSNP, the 1k human genome, the ESP6500 and BGI in house databases. Sanger sequencing confirmed the WES results. The proband carried the mutation homozygously, and unaffected parents were found to be heterozygous for the mutation (Fig. 2).

## DISCUSSION

PELD is a severe neurodegenerative disorder characterized by developmental regression of motor and cognitive skills in the first years of life and often results in patient’s death in the first decade of life. Clinical features include psychomotor regression, loss of speech, poor motor coordination with increased muscle tonicity, spasticity, ataxia, tremor, myoclonus-dystonia and seizures. A mild lipodystrophic phenotype with a lipoatrophic appearance can be found in most of the patients. Some of the affected people may have hypertriglyceridemia and hepatomegaly[20]. The Iranian patient had almost all of these clinical findings, as mentioned above.

In this study, we presented a proband with neuro-developmental regression, in which extensive neurologic and metabolic evaluations were not sufficiently specific for the diagnosis. We decided to perform WES on the affected patient in search for homozygous and potentially damaging gene variants. Analysis of WES revealed a known homozygous Sanger validated nonsense mutation c.985C>T (p.R329X) in exon 7 of the *BSCL2* gene related to PELD.

Based on the deleteriousness of the variant and the overlap between clinical features of the Iranian patient with the patients in whom the mutation was originally found, this variant was considered to be the most likely pathogenic candidate variant of the ones identified by WES. The variant is segregating in the family. PELD is an extremely rare condition; only six cases have been reported in scientific literature so far[20] and, to our knowledge, this is the first report of a PELD patient from Iranian population.

Identification of pathogenic mutation in rare Mendelian disorders could increase the quality of clinical diagnosis, patients management, and family counseling[23]. However, the establishment of a molecular diagnosis is particularly difficult in case of rare syndromes, in diseases with very high degrees of genetic and/or clinical heterogeneity and when clinical information in patients is limited or unspecific to reach to a conclusive diagnosis. Multiple studies have shown that WES technology can be beneficial to delineate the cause of the disease at DNA level and expedite the process of final clinical decision-making in situations of this nature[24,32].

In the present study, we have elucidated the genetic cause of PELD in an Iranian patient. We have also demonstrated the usefulness of WES in identifying the causative variant. This study illustrates how WES can be used in a clinical setting to identify pathogenic mutations in search for a specific diagnosis. Furthermore, WES can be used to accelerate clinical investigations of heterogeneous Mendelian traits as yet inconclusive cases.
